# Developing a Women’s Health track within addiction medicine fellowship: reflections and inspirations

**DOI:** 10.1186/s13722-022-00357-8

**Published:** 2023-01-09

**Authors:** Jordana Laks, Alexander Y. Walley, Sarah M. Bagley, Cecily M. Barber, Jessie M. Gaeta, Linda A. Neville, Alyssa F. Peterkin, Emily Rosenthal, Kelley A. Saia, Zoe M. Weinstein, Miriam T. H. Harris

**Affiliations:** 1 Grayken Center for Addiction, Section of General Internal Medicine, Clinical Addiction Research and Education Unit, Boston Medical Center, Boston University School of Medicine, 801 Massachusetts Avenue, 2nd Floor, Boston, MA 02118 USA; 2 Boston Health Care for the Homeless Program, Boston, MA USA; 3 Division of General Pediatrics, Department of Pediatrics, Boston University School of Medicine, Boston, MA USA; 4 Department of Obstetrics and Gynecology, Boston University School of Medicine, Boston, MA USA

**Keywords:** Addiction medicine fellowship, Women’s health, Medical education, Curriculum development

## Abstract

**Background:**

Women who use drugs face sexism and intersectional stigma that influence their drug use experiences and treatment needs. There is a need to build the capacity of addiction medicine specialists who can deliver gender-responsive services and advance research and policy in women-focused addiction care. We describe the development of a Women’s Health track within an addiction medicine fellowship program and reflect on successes, challenges, and future directions.

**Main body:**

The Women’s Health track was developed in collaboration between program leaders in Addiction Medicine and Obstetrics/Gynecology. Implementing the track led to the development of women-focused rotations and continuity clinics, as well as enrichment of women’s health didactic education for all fellows. The fellowship track spurred interdepartmental mentorship and collaboration on research and advocacy projects.

**Conclusion:**

Addiction medicine fellowships can replicate this curriculum model to advance women-focused education, research, and policy. Future curricula should focus on structural sexism in drug use and addiction treatment throughout a woman’s life course.

## Background

Women who use drugs engage in communities and social systems that reinforce gender-based inequities. While rates of substance use disorder (SUD) and drug overdose are rising more rapidly in women than men [1, 2], women are less likely to seek and receive SUD treatment [3, 4]. Male domination within relationships, drug-using communities, and treatment settings results in violence and coercion for women who use drugs. Intersectional oppression further marginalizes women based on their race, class, gender, and sexual identity [5].

Despite the increasing prevalence and harms of substance use in women, addiction services and research have traditionally been tailored to men or designed with gender-neutral approaches that fail to consider women’s specific needs [6]. Similarly, there is a gap in addiction education that prioritizes teaching on gendered dynamics of addiction and intersectional oppression of women who use drugs.

Addiction medicine fellowship programs, which are expanding to meet the national need for addiction care leaders, are optimally positioned to train women’s health addiction specialists. Dedicated curricula, funding, and mentorship are needed to train addiction medicine physicians who can advance women-focused addiction care, research, and policy. To address this gap, we developed a Women’s Health Track within an ACGME-accredited addiction medicine fellowship. In this commentary, we describe the impetus for the curriculum, development and implementation of the track, and reflections on future directions to enhance education on women’s health, gender, and sexism within an addiction medicine fellowship.

## Identifying the need: sexism and gender in substance use

Assessing the needs of women who use drugs requires a comprehensive understanding of gender and intersectionality. Gender refers to socially constructed roles that vary based on time and place, and gender identity reflects one’s internal sense of being a woman, man, or anywhere along the gender spectrum, including transgender, non-binary, and genderqueer identities. In this article and in the fellowship track, we define ‘women’ as all individuals who identify as a woman, regardless of their sex (classification as male or female based on biological attributes). Intersectional perspectives recognize that women’s experiences with drug use are not homogeneous. Rather, other intersecting identities, such as gender identity, sexual orientation, race/ethnicity, and socioeconomic class shape individual experiences of oppression or empowerment [5]. In particular, structural racism, homophobia, and transphobia enhance discrimination and treatment barriers for Black, Indigenous, and other racialized individuals and for transgender and genderqueer individuals compared to White cis-gender women who use drugs.

Women who use drugs interact with individuals, communities, and social systems that reproduce structural sexism. Structural sexism is defined as “discriminatory beliefs or practices on the basis of sex and gender that are entrenched in societal frameworks and which result in fairly predictable disparities in social outcomes related to power, resources, and opportunities” [7]. For example, gender-based power dynamics in drug-using communities may restrict women’s autonomy to determine when, how, and why they use drugs. Such power imbalances are associated with greater adverse consequences in women compared to men including higher rates of injection drug use-associated infections, co-occurring mood and anxiety disorders, and experiences of intimate partner violence and sexual exploitation [8, 9].

Structural sexism is also apparent in the systems that affect pregnant and parenting people who use drugs. Pregnant individuals who use drugs face punitive consequences from legal and child welfare systems, hostility from the general public, and an addiction treatment system that is poorly suited to meet their needs. The child welfare system has traditionally viewed prenatal and parental substance use as synonymous with abuse or neglect, causing heightened shame, stigma, and fear of seeking treatment. Black and Indigenous women are disproportionately harmed by trauma related to child welfare service reporting and custody loss.

The reality of structural sexism means that some women’s addiction care needs differ from those of men. Care, as discussed here, refers to a wide breadth of harm reduction services, addiction and mental health treatment, and medical care for people who use drugs. For example, communication about drug use experiences should account for inequitable relationships that may reinforce women’s drug use and/or present barriers to recovery. Sexual and reproductive health needs often intersect with women’s drug use and therefore should be addressed within addiction care settings. Additionally, addiction providers must work to mitigate systemic racism that minoritized women face in medical settings by using trauma-informed and racially sensitive approaches to care.

*Gender-responsive care* attends to how being a woman affects women’s experiences with substance use through its setting, staff, and services [10]. Addiction care tailored to women has demonstrated benefits, including increased treatment completion and treatment satisfaction, decreased use of substances, and reduced mental health symptoms. Despite this evidence, availability and access remain limited in the United States.

Prior to developing the Women’s Health track, our fellowship curriculum featured training experiences in perinatal care for women with SUD, but generally lacked training in structural sexism and its effects on women’s drug use and care needs. Additionally, service gaps remained within our institution and local treatment environment, particularly for Black and Hispanic women, gender minority groups, and non-pregnant or postpartum women. Thus, we aimed to create a fellowship track that would integrate and expand upon existing services and train physician leaders who could transform addiction care to work better for women.

## Developing the track

Growing enthusiasm for interdepartmental collaboration drove the development of the Women’s Health track and coincided with a funding opportunity from a donor interested in addiction care for women. The donor agreed to fund a one-year Women’s Health-focused position in our ACGME-accredited addiction medicine fellowship program. Financial support allowed the program to recruit fellows with a special interest in women's health (including authors MTHH and JL) who helped develop and enact the curriculum. For the academic year 2021–2022, the General Internal Medicine and Obstetrics and Gynecology (OB/GYN) departments collaborated to recruit the first joint Maternal Health Addiction Medicine fellow (author CB) to a two-year training program that included the existing curriculum adapted for an OB/GYN addiction medicine specialist.

We defined Women’s Health track core competencies based on the biopsychosocial model to prepare fellows to address biological, psychological, social, and systems issues affecting women who use drugs (Table 1). These competencies prioritize an understanding of how sexism manifests in drug use and addiction care environments and how this oppression is enhanced by other forms of discrimination based on race, class, language, immigration status, and other identities. The competencies also assert the responsibility of addiction specialists to promote gender equity in addiction care.

**Table 1:** Core competencies of the addiction medicine Women’s Health fellowship track

ACGME competency domain	Women’s Health track competencies
Patient Care	Provide trauma-informed, gender-responsive care to meet the needs of women who use drugs, including reproductive health, sexual health, and mental health needs
Medical Knowledge	Have the biomedical and clinical knowledge to care for women who use drugs, including knowledge on substance use disorder treatment for pregnant and parenting persons, contraceptive counseling, prevention of HIV, intimate partner violence, and sex work
Practice-based Learning and Improvement	Develop clinical expertise in caring for women with SUD through direct practice and guided practice improvement using clinical resources, including primary and secondary literature, professional conferences, and clinical guidelines
Interpersonal and Communication Skills	Counsel patients and their families on issues related to women’s health and addiction using a trauma-informed approach Collaborate in team-based models of care with other health care professionals, including psychiatry, obstetrics, and pediatrics providers
Professionalism	Demonstrate a commitment to carrying out professional responsibilities and adherence to ethical principles with sensitivity to a diverse and vulnerable patient population and dedication to promoting equity in patient care and professional activities Appreciate and ask about dimensions of identity that affect patients’ substance use and treatment experiences, including sex, gender identity, sexual orientation, race, class, immigration status, and religion Proactively work to reduce stigma against people who use drugs on an individual and institutional basis, with special attention to discrimination faced by women who use drugs
Systems-based practice	Identify and utilize systems of care for women who use drugs, including family-based addiction treatment programs and community-based harm reduction, housing, and other social service organizations that serve women Demonstrate an understanding of systems that disproportionately impact people who use drugs, including the child welfare, criminal-legal, mental health treatment, homelessness and housing systems Identify structural racism in addiction treatment and strategies to combat racism and promote equity in their practice and institution

ACGME: Accreditation Council for Graduate Medical Education

Educational strategies were split into three categories: clinical rotations; didactic curriculum; and research, QI, and advocacy. We increased the time fellows spent in existing programs designed for pregnant and parenting patients while creating new women-focused clinical opportunities in both academic and community settings. We also developed continuity clinic options in which fellows would practice women’s health clinical skills and build a patient panel of women with SUD. Fellows used elective time to explore other women-focused treatment settings, including a family-based residential treatment facility and the OB/GYN family planning clinic to gain experience in contraception and abortion care. The two-year Maternal Health Addiction fellowship schedule incorporated the same core addiction clinical rotations but also included specialized training in complex perinatal care, family planning including abortion procedures, and general gynecologic practice for patients with SUD.

For didactic education, we selected 13 key topics (Box 1) to include in the fellowship lecture schedule based on pivotal reviews on women and drug use. We also developed a list of clinical guidelines and online resources to help fellows enrich their education through self-directed learning (Box 1). The number and variety of didactics dedicated to women-focused issues increased after implementation of the Women’s Health track during the 2019–2020 academic year (Table 2). New presentations on sex work, intimate partner violence, trauma-informed care, and contraception and abortion enhanced the education of all fellows and built connections with new clinical and research experts.

**Table 2:** Fellow conference topics relevant to women’s health before (2018–2019) and after (2019 onward) developing the Women’s Health track

AY 2018–2019	AY 2019–2020	AY 2020–2021	AY 2021–2022
Managing OUD in pregnancy	Managing OUD in pregnancy	Managing OUD in pregnancy	Managing OUD in pregnancy
NOWS	NOWS	NOWS	NOWS
HIV prevention in PWID	HIV prevention in PWID	HIV prevention in PWID	HIV prevention in PWID
	Sex Work and SUD	Intimate Partner Violence in People who use Drugs	Intimate Partner Violence in People who use Drugs
		Fetal alcohol spectrum disorders	Fetal alcohol spectrum disorders
		Principles of trauma-informed care	Naltrexone for AUD and OUD in pregnancy
			Contraception and abortion for people with SUD
			Toxicology testing with patients monitored by social services

AY: academic year; OUD: opioid use disorder; NOWS: neonatal opioid withdrawal syndrome; HIV: human immunodeficiency virus; PWID: persons who inject drugs; SUD: substance use disorder; AUD: alcohol use disorder

Research, QI, and advocacy projects were supported by thoughtful connections to mentors. The first Women’s Health track fellow built professional relationships with nascent addiction medicine researchers resulting in multiple publications from research started during fellowship. The second fellow integrated into a multidisciplinary community health center team through her shelter-based continuity clinic and contributed to a QI project on screening for sexual health and contraception needs. The Maternal Health Addiction fellow led an initiative to provide rapid access to long-acting reversible contraceptives (LARCs) to hospitalized patients on the Addiction Consult Service and patients seen in a low-barrier SUD bridge clinic.

Fellow-led case conferences explored clinical and ethical issues in addiction treatment for women who use drugs. The Maternal Health Addiction fellow presented a case conference exploring challenges in OUD treatment for postpartum and parenting patients. Another case conference led to collaboration between the Pediatrics, OB/GYN, and Addiction Medicine departments on advocacy to change institutional and state policies governing mandated reporting of substance-exposed newborns.

Box 1. Core educational topics and learning resources on women and substance use

1. Understanding sex, gender, and gender differences in drug use
2. Structural sexism and intersectionality
3. Substance use in adolescents and young adults, including girls under age 18
4. Substance use in transgender, non-binary, and genderqueer populations
5. Perinatal treatment of SUD, including management of OUD
6. Neonatal withdrawal syndromes
7. Child welfare system involvement in people with SUD
8. Contraception and abortion for people who use drugs
9. Gender-responsive care
10. Sex work and substance use
11. Intimate partner violence and substance use
12. Trauma-informed care for women who use drugs
13. Co-occurring SUD and psychiatric disorders

**Self-directed learning resources:**

**Educational activities and practice resources:**

- ASAM eLearning Center: https://elearning.asam.org/
- ACAAM Education Portal: https://acaam.mclms.net/en/
- Providers Clinical Support Systems: https://pcssnow.org/education-training/
- Partners in Contraceptive Choice and Knowledge: https://picck.org/
- UCSF Transgender Care & Treatment Guidelines: https://transcare.ucsf.edu/guidelines
- National Center on Domestic Violence, Trauma & Mental Health: http://www.nationalcenterdvtraumamh.org/
- Sex Workers Outreach Project USA: https://swopusa.org/
- National Harm Reduction Coalition: https://harmreduction.org/resource-center/

**Clinical guidelines:**

- SAMHSA: Clinical Guidance for Treating Pregnant and Parenting Women With Opioid Use Disorder and Their Infants. January 2018. https://store.samhsa.gov/product/Clinical-Guidance-for-Treating-Pregnant-and-Parenting-Women-With-Opioid-Use-Disorder-and-Their-Infants/SMA18-5054
- SAMHSA: A Collaborative Approach to the Treatment of Pregnant Women with Opioid Use Disorders. 2016. https://store.samhsa.gov/product/A-Collaborative-Approach-to-the-Treatment-of-Pregnant-Women-with-Opioid-Use-Disorders/SMA16-4978

ASAM: American Society of Addiction Medicine; ACAAM: American College of Academic Addiction Medicine; SAMHSA: Substance Abuse and Mental Health Services Administration; UCSF: University of California, San Francisco.

## Reflections and future directions

Implementing the Women’s Health track enriched the training of all fellows by increasing the depth and range of women-focused clinical rotations and by reinforcing collaborations between OB/GYN and Addiction Medicine. Participating in the track has substantially influenced fellows’ career trajectories, leading to clinical roles, research projects, and teaching opportunities focused on improving care for women within the same local treatment environment. Ongoing benefits of the Maternal Health Addiction fellowship include strengthening rotations within the OB/GYN and Pediatrics departments, improving access to sexual and reproductive health care in addiction care settings, and building momentum for an interdepartmental community of women-focused addiction specialists who conduct research and teach together.

Systems-level facilitators to developing the fellowship track included previous collaboration between Addiction Medicine and OB/GYN educators on fellowship rotations, like the perinatal medical home for patients with SUD. Program leaders connected fellows to women’s health clinician-educators within the institution and in affiliated community health centers who were not historically involved in the fellowship but were willing to supervise or support fellows in new settings (for example, developing a continuity clinic in a women’s shelter-based clinic and shadowing in family planning clinic). Finally, the program manager (LAN) was instrumental in organizing a rotation schedule spanning multiple departments with different scheduling needs, particularly for the Maternal Health Addiction Fellowship that incorporated Labor & Delivery on-call time.

Future directions to improve the Women’s Health track include (1) improving existing, but siloed, clinical programs for families affected by substance use, (2) creating clinical rotations in women-only and family-based residential treatment programs, and (3) increasing opportunities to work with groups currently underrepresented in care, including Black and Hispanic women. We also strive to address medical and social issues affecting women across the life course, recognizing that our current curriculum disproportionately focuses on reproductive care. Incorporating foundational teaching on structural sexism and how it manifests in interpersonal relationships, drug-using communities, addiction care, and social systems is essential.

One potential criticism is that creating a Women’s Health track reproduces structural sexism by separating women’s health from mainstream education. However, we found that this process increased investment in women-focused education in a manner that would be difficult to achieve without a focused track. Moreover, all fellows, not just the Women’s Health track fellows, benefited from improved training on topics relevant to women who use drugs. In our current world, establishing a specialized fellowship pathway is a promising model to both train women-focused addiction specialists to design and deliver gender-responsive care and to move the whole field toward achieving greater gender inequity in addiction treatment and research.

The expansion of the Women’s Health track to include the Maternal Health Addiction fellowship designed for OB/GYN physicians reflects both progress and unmet need. There is great need for OB/GYN providers who can integrate addiction treatment into gynecologic and reproductive health care and lead research and policy changes to support pregnant people who use drugs. However, this expansion further shifts the educational focus toward women’s sexual and reproductive roles. Addressing non-reproductive issues for women (e.g., gender-based violence and trauma, infectious disease prevention and treatment) is also important. In truth, we need an expansion of women’s health addiction medicine providers across multiple specialties to care for women throughout their life course.

## Conclusions

Our experience shows that specialized women’s health training within an ACGME-accredited addiction medicine fellowship program is feasible and valuable, both as a curriculum track as well as a collaborative OB/GYN-Addiction Medicine Maternal Health Addiction fellowship. Other programs can use our experience as a roadmap to create or enhance women-focused clinical training, education, and mentorship. Expanding fellowship women’s health training will help prepare a new generation of addiction medicine leaders who can transform addiction care, research, and policy to better meet women’s needs.

## Data Availability

Not applicable.

## Abbreviations

ACGME: Accreditation Council for Graduate Medical Education
LARC: Long-acting reversible contraceptive
OB/GYN: Obstetrics and Gynecology
OUD: Opioid use disorder
QI: Quality improvement
SUD: Substance use disorder
